# Small understory trees increase growth following sustained drought in the Amazon

**DOI:** 10.1111/nph.70873

**Published:** 2026-01-02

**Authors:** Mateus C. Silva, David C. Bartholomew, André L. Giles, Paulo R. L. Bittencourt, Pablo Sanchez‐Martinez, Lion R. Martius, Vanessa N. Rodrigues, Rachel Selman, João P. Reis, Grazielle S. Teodoro, Rafael S. Oliveira, Oliver Binks, Maurizio Mencuccini, João A. Silva Junior, Antonio C. L. da Costa, Patrick Meir, Lucy Rowland

**Affiliations:** ^1^ Department of Geography, Faculty of Environment, Science and Economy University of Exeter Exeter EX4 4RJ UK; ^2^ Botanic Gardens Conservation International Richmond TW9 3BW UK; ^3^ Federal University of Santa Catarina (UFSC) Florianópolis 88040‐900 Brazil; ^4^ National Institute for Amazonia Research (INPA) Manaus 69067‐375 Brazil; ^5^ School of Earth and Environment Sciences Cardiff University Cardiff CF10 3AT UK; ^6^ School of GeoSciences University of Edinburgh Edinburgh EH9 3FE UK; ^7^ Geosciences Institute Federal University of Pará (UFPA) Belém 66075‐110 Brazil; ^8^ Institute of Biology State University of Campinas (UNICAMP) Campinas 13083‐970 Brazil; ^9^ Centre for Ecological Research and Forestry Applications (CREAF) Cerdanyola del Vallès 08193 Spain; ^10^ Catalan Institution for Research and Advanced Studies (ICREA) Cerdanyola del Vallès 08010 Spain

**Keywords:** acclimatisation, Amazonia, carbon metabolism, climate change, competition, phenotypic plasticity, plant hydraulics, rainfall regime

## Abstract

- Droughts pose a major threat to the Amazon rainforest, yet the mechanisms enabling trees to maintain growth under prolonged drought remain poorly understood, particularly in the understory layer.
- We leveraged a 22‐yr Throughfall Exclusion (TFE) in a 1‐ha plot in eastern Amazonia, paired with a Control plot, to test whether small understory trees (1–10 cm diameter) grow faster under long‐term drought due to acquisitive resource‐use strategies and competition release, given that the TFE plot experienced large‐tree mortality and canopy gap formation over time.
- Despite a 51% reduction in density, small trees grew 2.2 times faster in the TFE than in the Control. At the species scale, growth rates increased with acquisitive traits, such as high foliar nutrient concentrations, greater hydraulic conductivity, and higher leaf‐to‐wood area ratio, but only in the TFE. These shifts towards acquisitive resource‐use strategies were observed within species, indicating plastic responses to drought. At the community scale, growth rates were negatively associated with neighbour density in the TFE, suggesting that competition release facilitates growth under drought.
- Our findings reveal that plastic and competitive processes stabilise the growth of surviving small understory trees after drought‐induced self‐thinning, highlighting key mechanisms that can enhance forest resilience to future climate extremes.

## Resumo

- Secas são uma grande ameaça à floresta amazônica, mas os mecanismos que permitem que árvores mantenham o seu crescimento durante secas prolongadas ainda são pouco compreendidos, especialmente no estrato do sub‐bosque.
- Nós utilizamos uma parcela de 1 ha na Amazônia Oriental submetida a 22 anos de Exclusão Experimental da Precipitação (TFE), juntamente com uma parcela Controle, para testar se árvores pequenas do sub‐bosque (1–10 cm de diâmetro) crescem mais rápido após longos períodos de seca devido a estratégias aquisitivas de uso de recursos e ao relaxamento da competição, considerando a mortalidade de árvores grandes e a formação de clareiras ao longo do tempo na parcela TFE.
- Apesar de uma redução de 51% em densidade, as árvores pequenas cresceram 2,2 vezes mais rápido na parcela TFE do que na parcela Controle. No nível da espécie, as taxas de crescimento aumentaram com características aquisitivas, como altas concentrações foliares de nutrientes, maior condutividade hidráulica e maior razão área foliar–área de madeira, mas apenas na parcela TFE. Essas mudanças rumo a estratégias aquisitivas de uso de recursos foram observadas no nível intraespecífico, indicando respostas plásticas à seca. No nível da comunidade, as taxas de crescimento foram negativamente associadas à densidade arbórea na TFE, sugerindo que o relaxamento da competição facilita o crescimento sob seca.
- Nossos resultados revelam que processos plásticos e competitivos estabilizam o crescimento de árvores sobreviventes do sub‐bosque após o auto‐desbaste induzido pela seca e destacam mecanismos que podem aumentar a resiliência da floresta frente a extremos climáticos futuros.

## Introduction

The Amazon basin holds the largest continuous tropical rainforest on Earth, accounting for 20% of the global aboveground carbon stock (Malhi *et al*., 2006; Friedlingstein *et al*., 2023) and *c*. 25% of all known vascular plant species within only 3% of the planet's land surface (Cardoso *et al*., 2017; Freiberg *et al*., 2020). Since 1900, anthropogenic glasshouse gas emissions have raised global temperatures by 1.1°C (IPCC, 2023), contributing to more frequent and intense drought events in the Amazon region (Marengo *et al*., 2008; Lewis *et al*., 2011; Espinoza *et al*., 2024). Episodic droughts, often linked to El Niño Southern Oscillation (ENSO) events, can increase tree mortality in Amazonia (Berenguer *et al*., 2021), driving carbon and biodiversity losses (Bennett *et al*., 2023; Prestes *et al*., 2024) and reinforcing climate feedbacks that amplify regional warming and reduce rainfall (Flores *et al*., 2024). However, many regions of the Amazon are projected to experience not only episodic but also prolonged droughts that could persist for several decades (Duffy *et al*., 2015; Baker *et al*., 2021). Developing a mechanistic understanding of how forests respond to chronic drought is therefore critical for predicting the long‐term resilience of Amazonian ecosystems in the face of climate change.

Current understanding of tree responses to long‐term drought is biased toward canopy trees, while understory trees remain largely overlooked. Large trees are globally vulnerable to prolonged water deficits, which is attributed to them having greater evaporative demand and exposure to thermal‐ and wind‐related stresses, and potentially higher hydraulic vulnerability (Phillips *et al*., 2010; Bennett *et al*., 2015; Olson *et al*., 2018; Liu *et al*., 2019; Fernández‐de‐Uña *et al*., 2023; Araújo *et al*., 2024; Nixon *et al*., 2025). Persistent drought can cause leaf and branch loss, and ultimately tree mortality, creating canopy gaps that increase light availability and vapour pressure deficit (VPD) in the lower forest layers (Limousin *et al*., 2012; Saatchi *et al*., 2013; Rowland *et al*., 2015; De Frenne *et al*., 2021). Small trees in the shaded rainforest understory rely on diffuse light and sun flecks for photosynthesis and tend to maximise growth near canopy gaps (Brown, 1996; Zhou *et al*., 2023), making them especially sensitive to changes in light and VPD environments triggered by drought‐driven canopy loss. Evidence suggests that a decade of soil drought increased growth in lower canopy trees (20–40 cm diameter), but not in upper canopy trees (> 40 cm diameter) (Rowland *et al*., 2015). Understory trees (< 10 cm diameter) play a key role in forest regeneration following disturbances such as drought (Alonso‐Rodríguez *et al*., 2022), yet it remains unclear whether sustained drought also promotes growth in this stratum and whether any observed changes result from species turnover, as drought can alter understory species composition (Slik, 2004).

A shift towards acquisitive resource‐use strategies could result in enhanced growth in the drought‐exposed understory. Fast‐growing individuals and species typically exhibit traits that maximise resource uptake and photosynthesis, such as abundant, nutrient‐rich, carbon‐cheap leaves (Shipley, 2006; Gibert *et al*., 2016; Poorter *et al*., 2018; Gray *et al*., 2019) and hydraulically efficient wood (Fan *et al*., 2012; Hietz *et al*., 2017), although often at the cost of greater drought vulnerability due to xylem embolism (Gleason *et al*., 2016; Yan *et al*., 2020; Oliveira *et al*., 2021). These trait syndromes are known to drive growth responses under both episodic and chronic drought in canopy trees (Rowland *et al*., 2021; Smith‐Martin *et al*., 2023). Exposure to 15 yr of throughfall exclusion and consecutive canopy gaps led to understory trees increasing their photosynthetic capacity, nutrient‐use efficiency, leaf area, and hydraulic conductivity, although embolism resistance remained unchanged (Bartholomew *et al*., 2020; Giles *et al*., 2022). If such trait changes result from phenotypic plasticity, understory trees may have a greater capacity for acclimation, potentially buffering growth under drought conditions and enhancing post‐drought forest recovery (Nicotra *et al*., 2010; Lloret *et al*., 2012). However, it is still poorly understood whether acquisitive traits and plastic responses to sustained drought translate into increased growth in the regenerating forest stratum.

In addition to trait adjustments, competition dynamics can shape how understory trees respond to long‐term droughts. Drought‐induced mortality can reduce neighbour density, alleviating competitive pressure by increasing per capita access to light, water, and nutrients (Suarez & Sasal, 2012; Facciano *et al*., 2023). In Amazon, 15 yr of throughfall exclusion has resulted in substantial large tree mortality and leaf area reductions, but soil moisture per unit of biomass in droughted plots has returned to near‐control levels after 22 yr due to self‐thinning (Sanchez‐Martinez *et al*., 2025), underscoring the role of competitive release for coping with long‐term droughts (Lloret *et al*., 2012; Gleason *et al*., 2017). More broadly, experimental forest thinning is known to reduce stand‐level transpiration and increase water availability for remaining individuals (del Campo *et al*., 2022; Wang *et al*., 2022), ultimately improving tree growth and forest resilience under drought (Gavinet *et al*., 2020; Sankey & Tatum, 2022; Tonelli *et al*., 2023). The density of neighbouring individuals reflects local resource availability and can therefore serve as a proxy for competitive pressure (Weigelt & Jolliffe, 2003). While reduced neighbour density and competition can enhance growth in Amazonian understory trees (Hérault & Piponiot, 2018), this hypothesis remains untested in the context of long‐term drought experiments, which are key to understanding how the next generation of Amazonian trees will respond to intensifying climate extremes.

In this study, we unveiled the drivers of small understory tree growth under long‐term drought in the Amazon. We took advantage of the world's longest‐running rainforest throughfall exclusion experiment established in 2002 in Caxiuanã, eastern Brazilian Amazon. A 1‐ha plot has received *c*. 50% less rainfall due to a system of rainout panels (TFE, Throughfall Exclusion), while an adjacent 1‐ha unmodified plot receives ambient rainfall (Control). For the first time, we monitored the stem increment of 884 small understory trees (1–10 cm diameter) from 2017 to 2024. We linked stem increment rates to 16 hydraulic and photosynthetic traits measured in a subset of individuals derived from previous studies (Bartholomew *et al*., 2020; Giles *et al*., 2022) and to tree density as an indicator of competition, an analysis not previously conducted. Our goal was to test three hypotheses:

1. Long‐term drought enhances small understory tree growth.
2. Acquisitive resource‐use promotes growth under drought.
3. Drought‐driven competition release boosts growth.

## Materials and Methods

### Study site

This study was carried out in the Caxiuanã National Forest, state of Pará, Brazil (1°44′14.082″S, 51°27′18.8892″W). In 2002, 1 hectare of *terra firme* lowland seasonal rainforest was covered with transparent plastic rainout shelters at 1–2 m height connected to a gutter system, resulting in *c*. 50% of throughfall interception (TFE plot, Supporting Information Fig. S1). A 1‐hectare Control plot was installed 10–20 m from the TFE plot. The periphery of both plots was trenched 50–150 cm deep to prevent lateral flow and drain the intercepted throughfall. The panels and gutters have been maintained since 2002 and are regularly adjusted to allow small trees to grow vertically and flipped to allow litterfall to reach the ground. Aboveground biomass and large tree density (> 10 cm DBH) were similar between plots in 2002 (Control: 488 Mg ha^−1^, 523 ind. ha^−1^; TFE: 496 Mg ha^−1^, 494 ind. ha^−1^) but declined in the TFE by 2023, while remaining relatively stable in the Control (Control: 513 Mg ha^−1^, 500 ind. ha^−1^; TFE: 331 Mg ha^−1^, 466 ind. ha^−1^) (Sanchez‐Martinez *et al*., 2025). Leaf area index (LAI) was initially similar between plots (*c*. 5.4) (Metcalfe *et al*., 2010) but declined in the TFE from year 2, remaining below the control until 2014 (Control, *c*. 5.7; TFE, *c*. 4.9) (Rowland *et al*., 2015). The mean annual temperature is 26°C and precipitation is 2092 mm, based on meteorological records from 1996 to 2023 at a station located in the Control plot (Fig. S2). The dry season lasts 4 months (Aug–Nov), defined as months with cumulative precipitation below 100 mm (Fig. S2). The predominant soil type is yellow oxisols (ICMBio, 2012), with a soil depth of 3–4 m and a water table located deeper than 4 m (Fisher *et al*., 2007; Markewitz *et al*., 2010).

### Forest inventory and growth calculations

Within each 1‐ha plot, we established 20 subplots of 100 m^2^ (10 × 10 m) in May 2017. The area excluded any subplots found within 10 m of the edge of the 1‐ha plots to minimise any edge effects of the trenches. Within this 4000 m^2^ area, we tagged all trees with a stem diameter at breast height (DBH, 1.3 m) falling between 1 and 10 cm, hereafter referred to as ‘small trees’. We define a tree as a freestanding woody plant excluding ferns and palms. We measured DBH with tape or digital callipers depending on diameter and identified the individuals at the species level by consulting herbaria specimens and experts. We remeasured the individuals in November 2017, February 2018, July 2018, January 2019, August 2019, October 2020, October 2021, July 2022, and April 2024. In each census, we recorded whether the individuals were alive or dead and stopped the measurements when the individuals grew beyond the inclusion criteria of DBH 1–10 cm. Recruits, that is, individuals who made it to the 1 cm inclusion criteria, were tagged and identified only during the last census (April 2024). We automatically reviewed all individuals with a DBH change greater than 1 cm between 2017 and 2024 and corrected tabulation errors where DBH values spiked tenfold before returning to normal (e.g. 1.1, 1.2, 1.3, 1.4, and 1.5). We estimated stem increment through the slope of the linear model fitted to DBH as a function of time using all available measurements per individual (Fig. S3). We fitted a model to each individual separately and reported the stem increment in centimetres per year. We further manually inspected all DBH time series with increment rates below 0 or above 1 cm yr^−1^, excluded 362 measurements showing DBH changes greater than 1 cm within less than a year (4.5% of all records), and recalculated the stem increment for the affected individuals. Recruits were not included in the growth analyses due to the lack of repeated DBH measurements.

### Functional traits measurement

We sampled 76 individuals spread across 19 species (DBH 1–10 cm) between August and September 2017 to measure functional traits (Table S1). Individual selection targeted the 12 most abundant genera shared between plots (*Duguetia*, *Protium*, *Licania*, *Inga*, *Swartzia*, *Vouacapoua*, *Ocotea*, *Eschweilera*, *Mouriri*, *Iryanthera*, *Minquartia*, and *Manilkara*) (Bittencourt *et al*., 2020; Rowland *et al*., 2020). The sampling totalled 43 individuals in the Control plot (18 species) and 33 in the TFE plot (14 species). We measured 16 functional traits in the selected individuals. To avoid damaging the individuals, we reused the same branch for multiple measurements whenever possible. One branch, *c*. 1 m in length, was collected between 09:00 and 10:00 h for CO_2_ response curves, leaf morphology, and nutrient concentration measurements. Immediately after cutting, the branch base was recut twice underwater and allowed to stabilise in full sunlight for 30 min. A second branch was collected between 04:00 and 06:00 h for predawn leaf water potential and embolism resistance measurements. Finally, a third branch was collected between 11:30 and 13:30 h for measuring midday leaf water potential, hydraulic conductivity, wood density, minimum conductance, and leaf‐to‐sapwood area ratio. For a detailed description of the functional trait measurements, see Bartholomew *et al*. (2020) and Giles *et al*. (2022).

#### CO_2_ response curves

We performed CO_2_ response curves (*A*/*C*_i_) in one fully expanded healthy leaf by recording steady‐state carbon assimilation rates (*A*) across a range of CO_2_ concentrations (400, 200, 75, 400, 800, 1200, and 2000 ppm) under photosynthetic active radiation (PAR) of 1500 μmol m^−2^ s^−1^, 28°C, and relative humidity (RH) of 60–70% (cross‐calibrated Licor 6800 and 6400XT). We estimated the maximum rate of Rubisco carboxylase activity (*V*_cmax_) and the maximum rate of photosynthetic electron transport (*J*_max_) at 25°C following Farquhar *et al*. (1980) C3 photosynthesis model. We estimated leaf dark respiration (*R*_dark_) in an adjacent leaf by measuring gas exchange three times in 5 s intervals after wrapping the leaf in aluminium foil for 30 min (400 ppm CO_2_, PAR of 0 μmol m^−2^ s^−1^, 28°C). We standardised *R*_dark_ to 25°C using a Q10 value of 2.2.

#### Leaf morphology

We rehydrated and scanned the two leaves used in the CO_2_ curves and measured the leaf blade thickness (*L*_th_) at three points using digital callipers and averaged per leaf. We determined the leaf area in ImageJ (Schneider *et al*., 2012), dried the leaves for 24 h at 70°C to obtain the dry mass, and divided the dry mass by leaf area to obtain the leaf mass per area (LMA).

#### Nutrient concentration

We removed the major vein, dried (24 h, 70°C), and ground additional leaves for the nutrient analysis. We used the Kjeldahl method (Stuart, 1936) to determine total nitrogen content (N_mass_) and the ammonium metavandate method (Varley, 1966) to quantify total phosphorus content (P_mass_). Leaf nutrient concentrations were normalised to leaf dry mass.

#### Leaf water potential

We measured predawn leaf water potential (Ψ_pd_) on the branch collected between 04:00 and 06:00 h, and midday leaf water potential (Ψ_md_) on the branch collected between 11:30 and 13:30 h, using a pressure chamber (PMS 1505).

#### Embolism resistance

We used the pneumatic method (Pereira *et al*., 2016, 2020) to determine the leaf water potential at 50% (P50) and 88% (P88) loss of hydraulic conductivity (expressed in negative values). We rehydrated the branch for 24 h and applied a vacuum to the cut end of the branch for 2.5 min while it dehydrated. We monitored changes in pressure inside the vacuum reservoir, which were converted to the volume of air discharged. We measured the water potential of one to two leaves before each air discharge to infer the wood xylem water potential. The air discharge and water potential measurements were made in intervals of at least 1 h, and the branch was sealed in black plastic bags for stabilisation in between the measurements. We relativised the air discharged to its maximum and fitted a sigmoid curve to the percentage of air discharged (PAD) vs water potential.

#### Hydraulic conductivity

We measured maximum xylem‐specific hydraulic conductivity (K_smax_) using the pipette method after flushing stems to remove emboli (Sperry *et al*., 1988). Native embolism was assessed as the per cent loss of conductivity (PLC), calculated from the conductivity before and after flushing. Midday branches were rehydrated and trimmed underwater to prevent cutting‐induced embolism (Venturas *et al*., 2015). Stem segments (30–55 mm long, 3–5 mm diameter) were recut underwater and connected to a Venturi apparatus (Tyree, 2002). Flow was calculated from pressure drop using pressure transducers (26PCCFA6G) and a data logger (OM‐CP‐VOLT101A). All procedures were performed submerged to avoid artefactual embolism.

#### Wood density

We calculated wood density (ρ) using the water displacement method (Pérez‐Harguindeguy *et al*., 2013). A 40–80 mm long debarked subsample was rehydrated for 24 h and submersed in water for volume calculation and dried at 60°C until constant mass and weighed for dry mass calculation.

#### Leaf minimum conductance

We measured leaf minimum conductance (*g*_min_) by leaving the leaf in the dark for 30 min and then coupling a Licor system on the abaxial surface (Duursma *et al*., 2019).

#### Leaf‐to‐sapwood area ratio

We determined the ratio between the leaf area and sapwood area (*A*_l_ : *A*_s_) by measuring the debarked diameter of the branch base with a digital calliper. We measured the total leaf area by scanning all the leaves attached to the branch in ImageJ.

### Density and basal area calculations

We calculated the density of small trees (DBH 1–10 cm) and large trees (DBH > 10 cm) from the 2024 census at the subplot level as a proxy for competition. The small tree data came from the census cited above, and the large tree data came from Sanchez‐Martinez *et al*. (2025) (Fig. S4). Density was calculated as the number of individuals per subplot. As a complementary metric, we calculated the basal area of the same individuals, defined as the sum of stem cross‐sectional areas per subplot and expressed in square metres per hectare. Note that we only use the large tree data for the subplots where we have small tree data available. We averaged the stem increment per subplot to integrate it with the density and basal area data.

### Statistical analysis

#### Vegetation characterisation

We characterised size distributions using histograms with 0.5 cm DBH bins for the initial community (2017), the final community (2024), and the individuals that died during the study, for which we used their 2017 DBH values. We performed a two‐way ANOVA to test whether variation in small tree abundance and species richness was explained by plot treatment (TFE vs Control), sampling year (2017 vs 2024), and their interaction at the subplot level.

#### Hypothesis 1 *–* Long‐term drought enhances small understory tree growth

We tested Hypothesis 1 by fitting mixed‐effects linear models to stem increment as a function of the plot treatment at the individual scale. We included the species nested within the genus as the random intercept effect. We estimated the marginal (*R* ^2^_m_, variance explained by fixed effects) and conditional (*R* ^2^_c_, variance explained by fixed and random effects) coefficient of determination. We performed three additional tests to get further insight into whether differences in growth might be driven by size or species turnover. First, we tested if initial DBH predicts individual stem increment with a mixed‐effects model (species nested within genus as the random intercept). Second, we split the species into ‘shared’, the ones common to both plots, and ‘exclusive’, the ones unique to a plot, and ran paired Student's *t*‐tests comparing the species‐averaged stem increment between TFE and Control individuals for shared species. Third, we ran Student's *t*‐tests comparing the average stem increment of shared and exclusive species within both the TFE and Control plots. Note that the t‐tests used paired samples, which is why species‐level averages were employed.

#### Hypothesis 2 – Acquisitive resource‐use promotes growth under drought

We first averaged stem increment rates and trait values per species within each plot to align the two datasets. Then, we conducted a Principal Component Analysis (PCA) on the 16 plot‐ and species‐averaged traits to identify major axes of trait variation. Only principal components (PCs) explaining more than 10% of the variance were retained, and their relationships with the 16 traits were assessed using Pearson correlation coefficients. To test Hypothesis 2, we fitted linear models of species mean stem increment as a function of the interaction between PCA axes and plot. We performed automated model selection, ranked models by Akaike Information Criterion (AIC), and selected the model with the lowest AIC. To assess whether growth–trait relationships differ between the plots due to intraspecific trait variation, we calculated the difference in stem increment (Δstem increment: stem increment_TFE_ − stem increment_Control_) and PC scores (ΔPC: PC_TFE_ − PC_Control_) between TFE and Control plots for shared species. We then fitted a linear model to Δstem increment as a function of ΔPC, again ranking models by AIC and retaining the best model (i.e. lowest AIC).

#### Hypothesis 3 – Drought‐driven competition release boosts growth

We tested Hypothesis 3 by fitting two linear models to the subplot average stem increment, one as a function of small tree density (1–10 cm DBH) and another as a function of large tree density (> 10 cm DBH). All models included an interaction term with plot. We further performed three additional analyses. First, we fitted similar models but with basal area as the response variable instead of density to better capture size‐dependent competition pressures. Second, we fitted an alternative, more complex model (formula: stem increment ~ (small tree density + large tree density) × plot) to account for joined effects of small and large tree densities as predictors of small tree increment rates. Third, we tested whether the density and basal area of small trees were correlated with those of large trees using Pearson's correlation.

We performed all the analyses on R v.4.4.1 (R Core Team, 2024). We used the function lmer from the package lme4 for fitting the mixed‐effects linear models (Bates *et al*., 2015) and functions ‘r.squaredGLMM’, ‘dredge’, and ‘model.avg’ from the package mumin for estimating *R* ^2^_m_ and *R* ^2^_c_, performing the model selection, and averaging the models, respectively (Bartoń, 2025). The data and code required to reproduce the analyses are available at doi: 10.5281/zenodo.17879009.

## Results

### Community structure, diversity, and dynamics

**Fig. 1:**
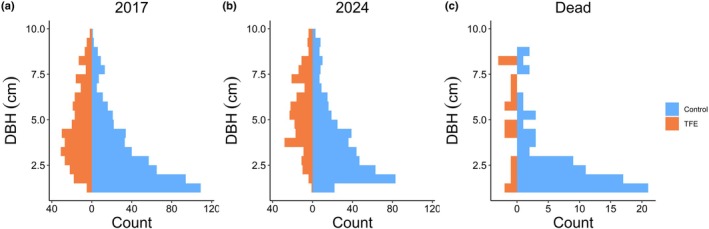
Size structure of the small tree community. Number of individuals per stem diameter (DBH) class in 0.2 ha (20 × 100 m^2^ subplots) in the Throughfall Exclusion (TFE) and 0.2 ha in the Control plot, showing (a) the initial census in 2017, (b) the final census in 2024, and (c) the subset of dead trees, with DBH referenced to 2017.

**Fig. 2:**
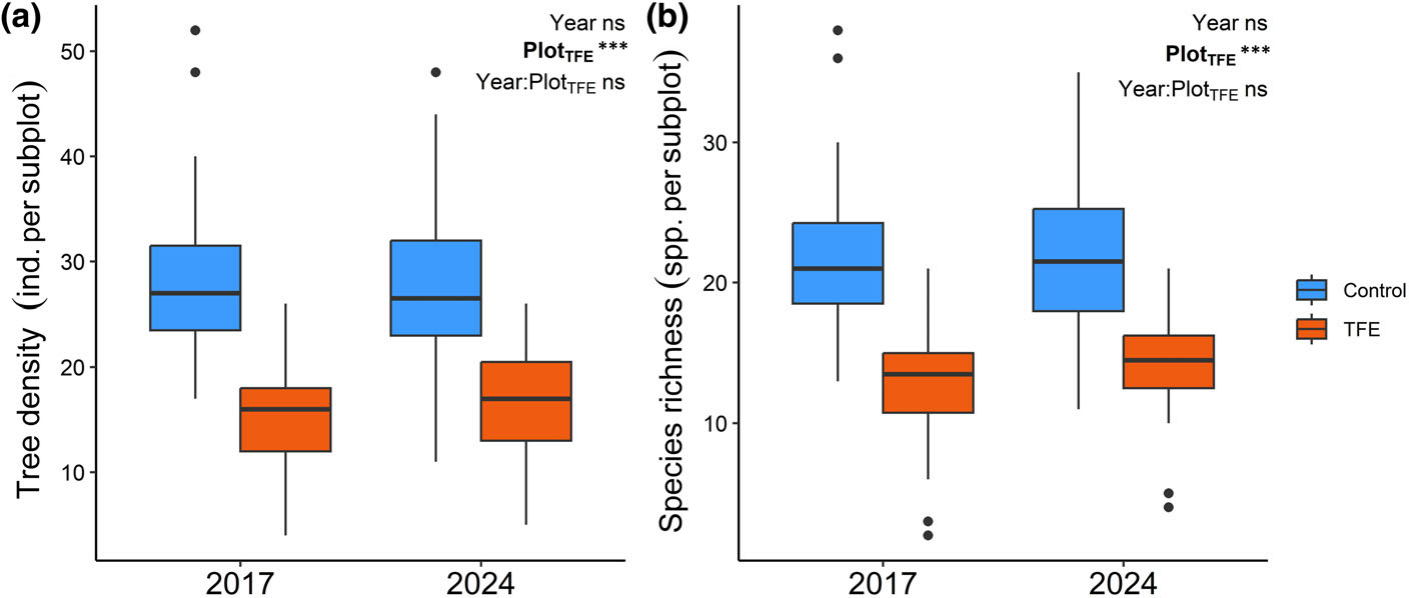
Drought effects on the understory structure and diversity. (a) Small tree density and (b) richness per subplot in the Throughfall Exclusion (TFE) and Control treatment. Each observation corresponds to a 100 m^2^ subplot (*n* = 20 per treatment) sampled in 2017 and 2024. *P*‐values at the top‐left corner correspond to two‐way ANOVA significance levels for the effect of year, plot, and year : plot interaction. ‘ind.’ stands for individual and ‘spp.’ for species. The horizontal line marks the median, the whiskers show the data range, and the points indicate outliers. Bold indicates statistically significant predictors (*P* < 0.05). Significance levels: ns or non‐significant, *P* > 0.05, ***, *P* ≤ 0.001.

We recorded 301 small trees (DBH 1–10 cm) in the TFE and 583 in the Control plot. Throughout the study, 94 out of the initial 884 individuals died, of which 17 (5.6%) were in the TFE plot and 77 (13.2%) in the Control plot. An additional 118 individuals met the inclusion criteria of DBH > 1 cm during the last census (April 2024), totalling 49 recruits in the TFE and 69 in the Control plot. When grouped by size classes, the Control was dominated by small stems, whereas the TFE showed a more symmetrical size distribution across the first (2017) and last (2024) censuses, as well as among dead individuals (Fig. 1). We identified 208 species (TFE: 125, Control: 172) representing 114 genera (TFE: 75, Control: 97) and 47 families (TFE: 35, Control: 42). The plots shared 90 species. *Protium*, *Pouteria*, and *Licania* were the most abundant genera in both plots. At the subplot level, TFE subplots had a lower small tree density and species richness than Control subplots (*P* < 0.001, Fig. 2). Neither the year (2017 vs 2024) nor the interaction between plot and year had a significant effect on individual density and species richness at the subplot level (*P* > 0.05 for all comparisons, Fig. 2).

### Growth responses to long‐term drought (Hypothesis 1)

**Fig. 3:**
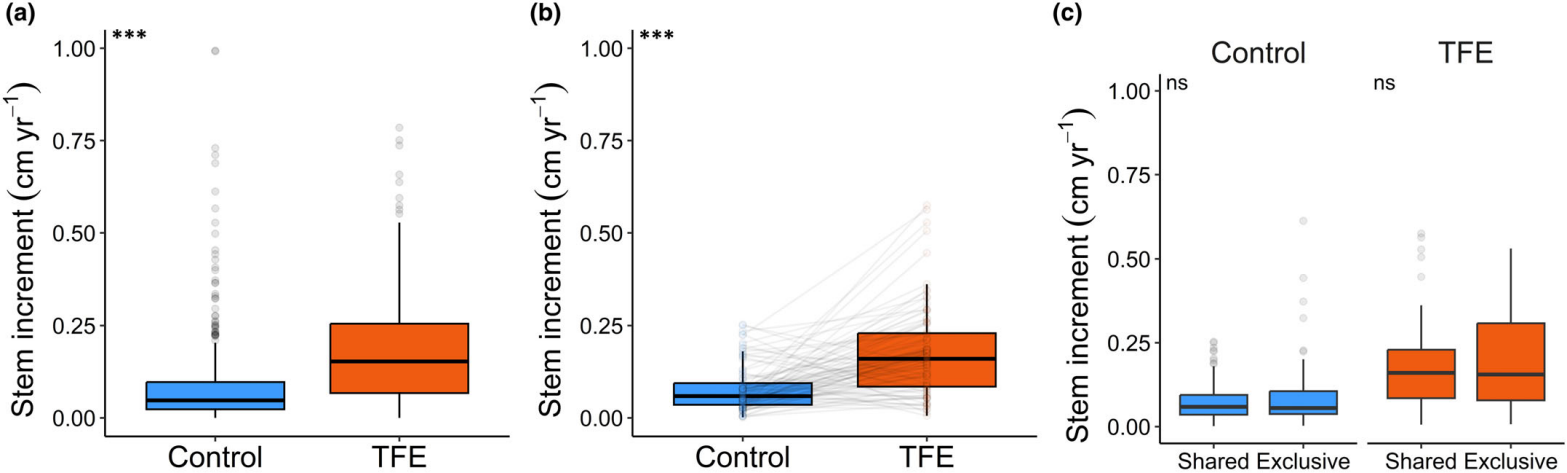
Long‐term drought effects on small tree growth rates. (a) Individual‐based stem increment estimated by the change in stem diameter at the breast height (DBH) from 2017 to 2024 for trees with DBH 1–10 cm in the Throughfall Exclusion plot (TFE) and Control plot. (b) Species‐averaged stem increment for shared species in the TFE vs Control plot and (c) for shared vs exclusive species within each plot. Shared species are found in both plots, and exclusive species are those that are singular to one plot. The line in panel b connects the same species. *P*‐values at the top‐left corner of panel a correspond to a mixed‐effects linear model fitted to stem increment as a function of plot, at panel b to paired *t*‐tests, and at panel c to *t*‐tests run for each plot separately. The horizontal line marks the median, the whiskers show the data range, and the points indicate outliers. *P* < 0.05 is in bold. Significance levels: ns or non‐significant, *P* > 0.05, ***, *P* ≤ 0.001.

Stem increment was higher in the TFE plot compared to the Control plot (*P* < 0.001, Fig. 3a). Small trees in the TFE plot increased DBH by a rate of 0.18 cm yr^−1^ (±0.14 SD) vs 0.08 cm yr^−1^ (±0.10 SD) in the Control plot, which represents a 125% or a 2.25‐fold increase. The fixed effects (plot, *R* ^2^_m_) explained 14.1% of the stem increment variability at the individual scale, whereas the fixed and random effects combined (plot + genus and species, *R* ^2^_c_) increased explanation power to 17.3%. Stem increment increased with initial DBH at the individual level, with a similar relationship in both plots (*P* < 0.001; Fig. S5). Stem increment also remained higher in the TFE plot compared to the Control plot after accounting for differences in species composition (Fig. 3b). Paired *t*‐tests between species occurring in both plots (shared) showed that the species mean stem increment was 122% higher in the TFE plot than in the Control plot (Fig. 3b). Species‐averaged stem increment did not differ between shared and exclusive (i.e. species occurring in only one plot) within either the TFE or Control plots (*P* > 0.05 for both comparisons, Fig. 3c).

### Drought effects on growth–trait associations (Hypothesis 2)

**Fig. 4:**
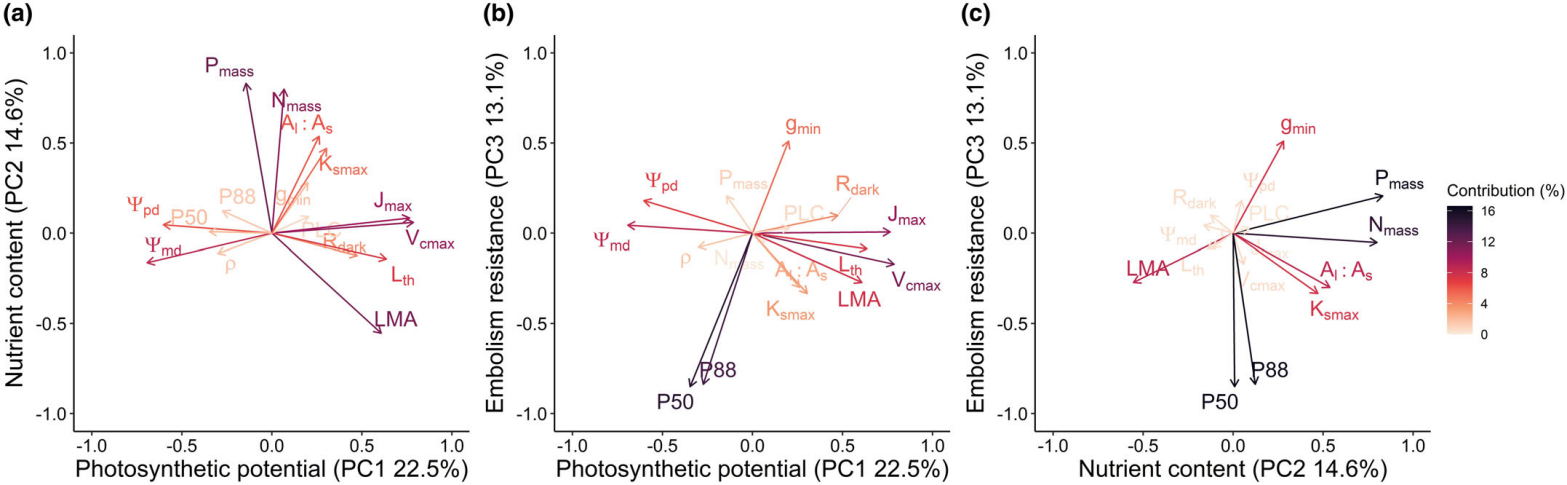
Trait covariation among small trees. Principal component analysis (PCA) of 16 metabolic and hydraulic traits. Only the first three principal components (PCs) were retained. Panel (a) show the relationship between PC1 and PC2, (b) PC1 and PC3, and (c) PC2 and PC3. PCs were interpreted as follows: PC1 ‘Photosynthetic potential’, PC2 ‘Nutrient content’, and PC3 ‘Embolism resistance’. Axis labels indicate the percentage of variance explained by each PC. Arrows represent the original traits in the PCA space, and colours indicate each trait's combined contribution to the two PCs shown in each panel, with warmer colours reflecting stronger influence. *V*_cmax_ corresponds to maximum rate of Rubisco carboxylation; *J*_max_, maximum electron transport rate; *R*_dark_, leaf dark respiration rate; *g*_min_, leaf minimum conductance; N_mass_, leaf nitrogen content; *P*_mass_, leaf phosphorus content; LMA, leaf mass per area; *L*_th_, leaf thickness; ρ, wood density; Ψ_pd_, predawn leaf water potential; Ψ_md_, midday leaf water potential; P50, water potential at which 50% of conductivity loss; P88, water potential at which 88% of conductivity loss; K_smax_, maximum xylem‐specific hydraulic conductivity; PLC, percentage loss of xylem conductivity; and *A*_l_ : *A*_s_, leaf‐to‐sapwood area ratio.

**Fig. 5:**
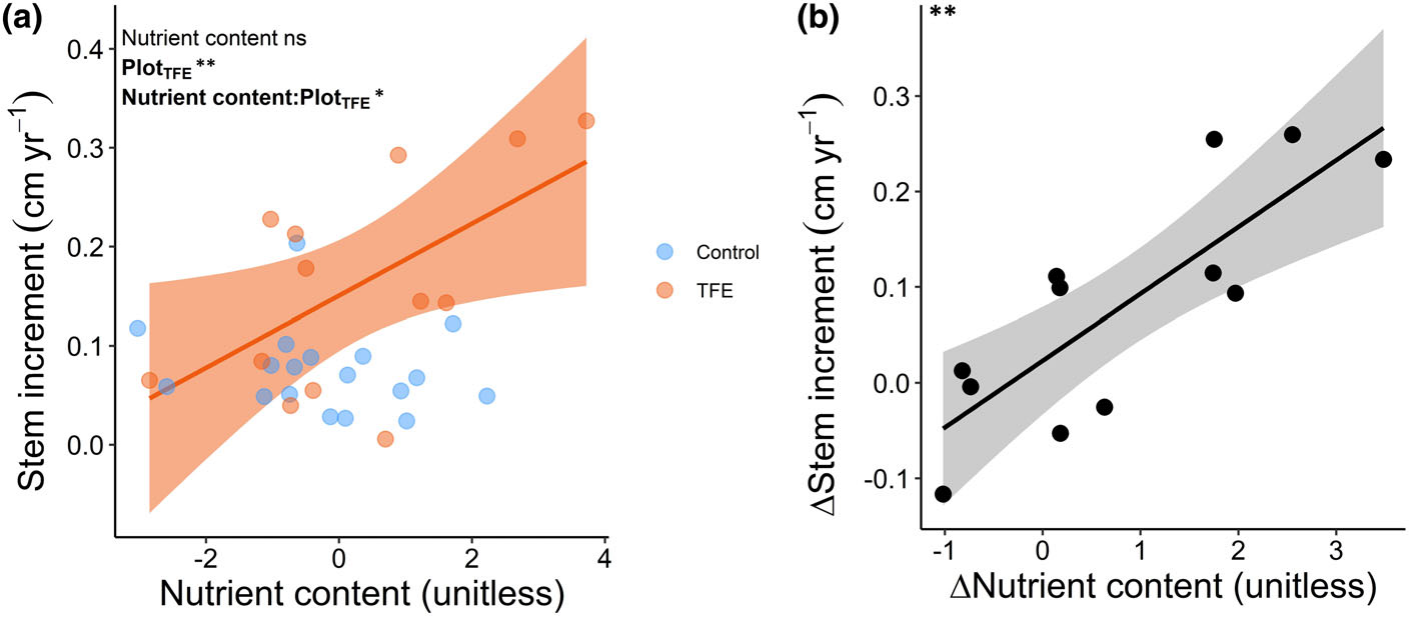
Functional drivers of small tree growth. (a) Relationship between stem increment per species and the nutrient content axis (PC2) in Throughfall Exclusion (TFE) and Control plots. (b) Relationship between the difference in stem increment (Δstem increment) between TFE and Control plots for a subset of shared species and the corresponding difference in nutrient content (Δnutrient content). Each point represents a species. *P*‐values in the top‐left corner correspond to the linear models fitted to (a) stem increment as a function of nutrient content, plot (TFE), and their interaction, and (b) Δstem increment as a function of Δnutrient content. Models shown are those with the lowest AIC following model selection. A colon ‘:’ denotes an interaction term. *P* < 0.05 values are shown in bold. Only statistically significant fitted lines are displayed. Shaded area shows the 95% confidence interval. Significance levels: ns or non‐significant, *P* > 0.05; *, *P* ≤ 0.05; **, *P* ≤ 0.01.

The first three principal component (PC) axes explained 50.1% of the variation across the 16 functional traits (Fig. 4, Table S2). PC1, interpreted as photosynthetic capacity, was positively correlated with *V*_cmax_, *J*_max_, *R*_dark_, LMA, and *L*_th_, and negatively with Ψ_pd_ and Ψ_md_. PC2, representing nutrient content, was positively correlated with N_mass_, P_mass_, K_smax_, and A_l_:A_s_, and negatively with LMA. PC3, reflecting embolism resistance, was positively correlated with g_min_ and negatively with P50 and P88. The best model revealed a positive effect of the TFE plot (*P* = 0.007) and a significant positive interaction between nutrient content and TFE (*P* = 0.03) on species‐averaged small tree stem increment (Fig. 5a; Table S3). Nutrient content (*P* = 0.63) and embolism resistance (*P* = 0.10) were retained in the model but had no significant overall effects on species mean increment. The best model for Δstem increment, fitted to a subset of 11 species shared between TFE and Control plots, included only Δnutrient content, which had a positive effect on Δstem increment (*P* = 0.001, Fig. 5b; Table S3).

### Growth–competition relationships under drought (Hypothesis 3)

**Fig. 6:**
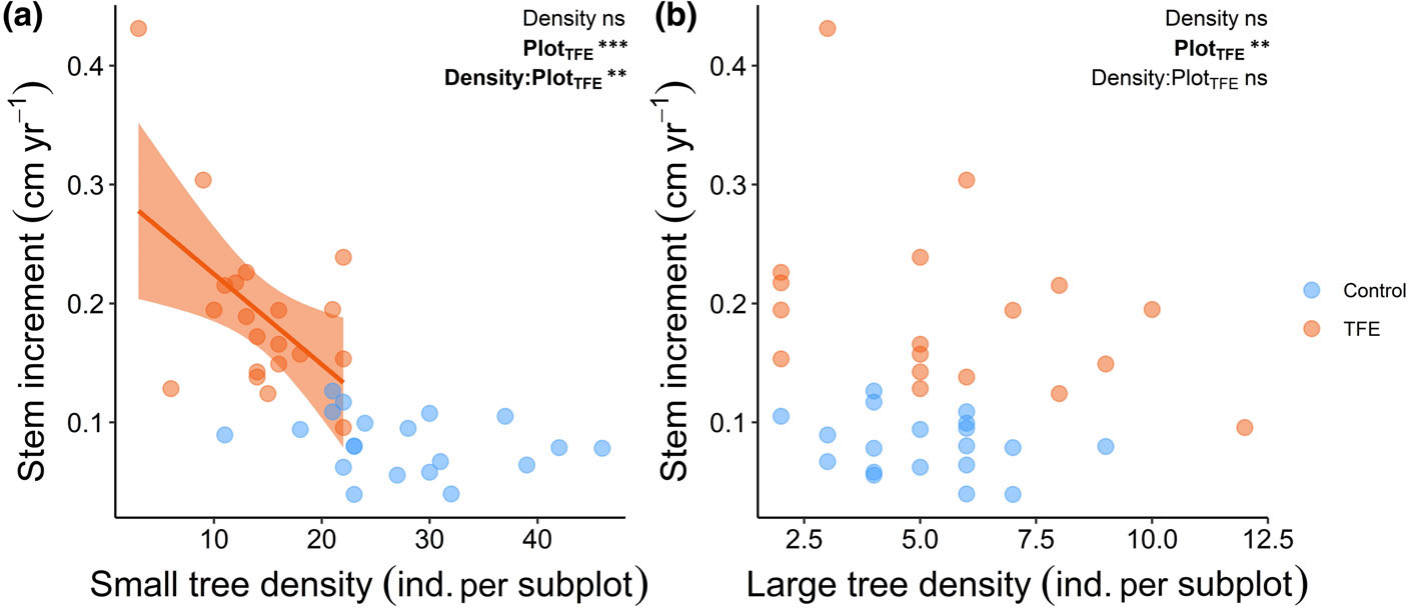
Effect of competition on small tree growth rates. (a) Relationship between small tree stem increment versus small tree density (DBH 1–10 cm) and (b) large tree density (DBH > 10 cm). Each observation corresponds to a subplot (100 m^2^) within the Throughfall Exclusion plot (TFE) and Control plot. Stem increment was averaged per subplot. ‘ind.’ stands for individual. *P*‐values at the top‐left corner correspond to the linear models fitted to the stem increment as a function of the density, plot (TFE), and their interaction. A colon ‘:’ denotes an interaction term. *P* < 0.05 is in bold. Only statistically significant fitted lines are shown. Shaded area shows the 95% confidence interval. Significance levels: ns or non‐significant, *P* > 0.05; **, *P* ≤ 0.01; ***, *P* ≤ 0.001.

The TFE treatment modulated the effect of small tree density on increment rates at the subplot level. When averaging across the subplot means, stem increment was 0.19 cm yr^−1^ (±0.07 SD) in TFE subplots and 0.08 cm yr^−1^ (±0.02 SD) in Control subplots. The small tree density had an overall non‐significant effect on stem increment at the subplot level (*P* = 0.60, Fig. 6a). However, the TFE plot affected stem increment positively (*P* < 0.001), and small tree density interacted with the plot to predict stem increment (*P* = 0.009). The negative interaction coefficient resulted in a stronger negative relationship between stem increment and small tree density in TFE subplots compared to Control subplots. Large tree density (Fig. 6b), as well as small and large tree basal area (Fig. S6), had no statistically significant effect on understory tree stem increment rates averaged per subplot, even considering the interaction effect with the plot (TFE vs Control). The alternative model, including both small and large tree densities as predictors, produced results qualitatively similar to the separate models (Table S4). Additionally, there was no correlation between the density and basal area of large trees vs the density and basal area of small trees within each plot (*P* > 0.05 for all comparisons, Table S5).

## Discussion

We investigated how 22 yr of experimental throughfall exclusion affected the growth rates of small understory trees in a lowland Amazon rainforest. Our findings indicate that sustained drought reduced small tree density and diversity but enhanced individual growth rates compared to ambient rainfall. On average, small trees grew two times faster in the TFE than in the Control. At the species level, higher growth in the TFE was associated with increased foliar nitrogen and phosphorus content, lower leaf mass per area, higher leaf‐to‐sapwood area ratios, and greater maximum xylem‐specific hydraulic conductivity. Shared species that shifted these traits toward a more acquisitive resource‐use strategy also achieved higher growth in the TFE relative to the Control. At the subplot level, small tree growth declined with increasing density of individuals in the understory layer, but this effect was evident only in the TFE plot. These results suggest that forests regenerating after prolonged drought may develop into low‐density tree assemblages characterised by more acquisitive resource‐use strategies, reflecting dynamics typical of secondary forests.

### Multidecadal drought increases growth rates in the understory layer

Our data demonstrate that trees grow faster in a forest understory shaped by two decades of drought (Hypothesis 1). In the two independent throughfall‐exclusion experiments, imposed drought led to large tree mortality and increased canopy openness, relaxing competition for light in the forest understory (Brando *et al*., 2008; Rowland *et al*., 2015). More light reaching the forest understory and less shading can enhance small tree photosynthesis, which can boost growth, especially if the water and nutrients available are sufficient to supply the density of individuals present (Poorter & Hayashida‐Oliver, 2000; Bartholomew *et al*., 2020; Rowland *et al*., 2020). Such increased light in the TFE plot likely reflects both early leaf and branch loss, starting 1 yr after the experiment started (Metcalfe *et al*., 2010; Rowland *et al*., 2018), and large‐tree mortality that peaked after 10 yr of throughfall reduction (Rowland *et al*., 2015; Sanchez‐Martinez *et al*., 2025). Prolonged drought not only reduced the density of large canopy trees, as previously reported, but also decreased the density and diversity of small understory trees, a pattern, to our knowledge, not previously documented for rainforests. Similarly, secondary forests regenerating after anthropogenic disturbances such as selective logging and wildfires often exhibit sparser, less diverse understory assemblages with higher growth rates, linked also to the death of large trees and canopy openings (Gerwing, 2001; Prestes *et al*., 2020; Bartholomew *et al*., 2024; Díaz‐Talamantes *et al*., 2025). The stability of small tree density and species richness during the study (2017–2024), together with the stability of stand biomass over the same period (Sanchez‐Martinez *et al*., 2025), suggests that two decades of extreme drought have shifted the forest understory toward a novel, fast‐growing, secondary‐forest‐like state that has remained stable for at least the past 7 yr of monitoring.

We did not find evidence that fast growth under drought is species‐specific or due to an artefact. More than half of the recorded species were exclusive to either the TFE or Control plot (57%), indicating marked species turnover. Previous studies have found that physiological responses to drought can be genus‐dependent (Binks *et al*., 2016; Bartholomew *et al*., 2020; Bittencourt *et al*., 2020; Giles *et al*., 2022). However, including genus and species in the model fitted to growth predicted by the drought treatment explained only 3.2% of additional growth variation. In fact, crown light exposure was also more important than genus in shaping tree growth (DBH > 10 cm) at this site, suggesting growth may depend more on light environment than on taxonomy (Poorter, 1999; Rowland *et al*., 2021). In our study, TFE small trees still grew faster than the Control ones when analysing only shared species and species found exclusively in the TFE specialists did not grow faster than those shared between the plots, suggesting the TFE treatment is not filtering for fast‐growing species. Additionally, the plastic panels could increase leaf temperature, VPD, and diffuse light for trees beneath them (Yahdjian & Sala, 2002), potentially causing very small trees (DBH 1–2 cm), whose canopies are mostly under the panels, to grow faster in the TFE as a panel artefact. However, stem increment increased with diameter similarly between plots, indicating no support for the panel artefact hypothesis. Therefore, fast growth is likely to be a resource‐driven, rather than a species‐specific or artefact‐related, response to drought.

### Acquisitive resource‐use underpins growth under prolonged drought

Supporting Hypothesis 2, traits associated with an acquisitive resource‐use strategy promoted faster growth in small understory trees under sustained drought. The fact that the nutrient content axis only influenced stem increment through an interaction with plot supports the idea that growth–trait relationships are context dependent (Yang *et al*., 2018). Under ambient rainfall, understory tree growth was unrelated to major axes of trait covariation (photosynthesis, nutrition, and hydraulics), consistent with observations elsewhere (Paine *et al*., 2015). Under drought, however, growth increased with the nutrient content axis, which reflects higher foliar nutrient concentrations (high N_mass_, P_mass_) and lower carbon investment per unit leaf area (low LMA) found in the small trees in the TFE. These trait combinations are associated with higher carbon assimilation and shorter leaf lifespan (Wright *et al*., 2004; Reich, 2014), a syndrome also common in early‐successional secondary tropical forests (Selaya & Anten, 2010; Lohbeck *et al*., 2015). While N_mass_, P_mass_, and LMA were the most influential traits, the nutrient content axis also correlated positively with the leaf‐to‐sapwood area ratio (*A*_l_ : *A*_s_) and branch maximum hydraulic conductivity (K_smax_). Therefore, fast growth in the TFE was not only supported by nutrient‐rich leaves but also by producing more foliage for a given water supply capacity (sapwood area) (Mencuccini *et al*., 2019) and increasing water transport efficiency (He *et al*., 2020). Surprisingly, PC1 did not influence stem increment, suggesting that leaf‐level photosynthetic potential may be a weaker growth predictor than leaf nutrients and *A*_l_ : *A*_s_, as previously reported for Australian rainforests (Gray *et al*., 2019). An integrated shift toward a more acquisitive resource‐use strategy under drought resembles patterns observed in secondary forests and likely results from increased light availability following drought‐induced canopy thinning.

The link between growth and acquisitive resource‐use in the droughted forest understory is likely driven by intraspecific trait variation. Community‐level results show substantial species turnover between the TFE and Control understories, potentially reflecting multiple years of drought selecting for species with inherently more acquisitive traits (Griffin‐Nolan *et al*., 2019; Yan *et al*., 2025). However, the ability to increase N_mass_, P_mass_, *A*_l_ : *A*_s_, and K_smax_ and decrease LMA, traits captured by the Δnutrient content axis, allowed individuals of the same species to grow faster in the TFE than in the Control, as reflected by higher Δstem increment. This positive Δstem increment–Δnutrient content relationship was unaffected by compositional turnover, as it was based exclusively on species shared between plots. Since most of the analysed species increased their Δnutrient content, the results indicate that intraspecific trait shifts underlie differences in growth–trait relationships across plots, independent of any effects of species filtering. Given that the TFE and Control plots are adjacent, with no barriers to gene flow, the observed variation in the nutrient content axis likely reflects phenotypic plasticity (Matesanz & Ramírez‐Valiente, 2019; Stamp & Hadfield, 2020), assuming that the interval between drought‐induced canopy opening (2013) and our trait sampling (2017) was probably too short for selection of high‐light‐adapted genotypes (Schmitt *et al*., 2025). In summary, fast growth under long‐term drought reflects directional intraspecific trait adjustment, most likely plastic responses, that promote a more acquisitive resource‐use strategy.

### Fast growth is linked to drought‐induced competition release

Weaker competition among similar‐sized individuals enhanced small understory tree growth, but only when exposed to a two‐decade‐long drought, partially supporting Hypothesis 3. The effect of neighbour density on growth was not detectable in the Control forest, potentially because Control subplots are likely light‐limited, as the dense canopy shades understory trees regardless of their density. However, long‐term drought alleviated light competition in the TFE understory through canopy gap creation (Suarez & Sasal, 2012; Rowland *et al*., 2015; Facciano *et al*., 2023), likely shifting the growth limitation away from light and more towards water and nutrients (de Costa *et al*., unpublished data). Combined with the imposed drought and drier soils, belowground competition for water and nutrients, many of which are taken up dissolved in water, likely becomes a key driver of growth (Schmied *et al*., 2023). This is supported by the trees in the drought plot investing more in water and nutrient uptake strategies, through increasing root allocation to deeper soil layers and increasing root exudation (de Costa *et al*., unpublished data). Given that understory trees have shallow roots and rely on near‐surface water (Brum *et al*., 2019), trees surrounded by fewer neighbours have more access to water and nutrients to sustain elevated levels of photosynthesis and growth in the light‐rich droughted forest.

Competition with larger canopy trees did not affect small understory tree growth, both in the TFE and Control forests. Although there is abundant evidence that competition is size‐dependent (Potvin & Dutilleul, 2009; DeMalach *et al*., 2016; Weng *et al*., 2022; Jo *et al*., 2025), we found no effect of the density and basal area of large trees (DBH > 10 cm) on the growth of small trees (DBH 1–10 cm) at the subplot scale. Two non‐exclusive hypotheses may explain this result. Our approach of inferring competition through individual density and basal area may be sufficient to capture competitive interactions between small‐statured trees, but too spatially restricted to capture the competitive effects of larger canopy trees on smaller understory trees. Canopy and emergent trees can affect the recruitment, growth, and survival of understory trees growing under their crowns through the shading effect (Valladares *et al*., 2016), but also over a much larger area covered by their rooting zones (Tumber‐Dávila *et al*., 2022), which we were not able to estimate. Our small trees are likely affected by larger trees beyond the subplot, on scales which are challenging to predict. Another possibility is that niche segregation exists across ontogenetic stages, reducing competition between canopy and understory trees. Canopy and emergent trees have deep roots and can access water across the soil vertical profile, while understory trees have shallow roots and rely on surface soil moisture (Brum *et al*., 2019). Therefore, drought effects on competition‐growth relationships may be more detectable and stronger among small‐sized individuals, as found here.

### Concluding remarks and future perspectives

We show that sustained drought promotes growth among surviving small understory trees in the Amazon rainforest, an understudied stratum that forms the foundation of forest regeneration. These growth responses are driven by a combination of plastic adjustments and competitive release, mechanisms previously proposed for canopy trees (Rowland *et al*., 2023; Sanchez‐Martinez *et al*., 2025) but, to our knowledge, not yet demonstrated for understory individuals. Under reduced competition and increased light availability from canopy openings, small trees adopt more acquisitive strategies, enabling faster growth. This acquisitive resource use and fast growth may allow understory trees to rapidly close canopy gaps, a response previously observed in disturbed forests (Craven *et al*., 2015). Yet, the concomitant loss of species richness may constrain biodiversity recovery even if forest structure eventually returns to pre‐drought levels, potentially trapping the forest in a secondary‐like state (Bartholomew *et al*., 2024). Our findings reveal that long‐term drought can reconfigure tropical forest understories into fast‐growing assemblages, with significant implications for forest regeneration and resilience following extreme climatic events.

## Competing interests

None declared.

## Author contributions

MCS and LR designed the study; DCB, ALG and LR collected the data; MCS processed the data and led the manuscript writing; PRLB, PS‐M, LRM, VNR, RS, JPR, GST, RSO, OB, MM, JASJ, ACLC and PM contributed to, reviewed, and approved the final manuscript.

## Disclaimer

The New Phytologist Foundation remains neutral with regard to jurisdictional claims in maps and in any institutional affiliations.

## Supporting information


**Fig. S1** Illustration of the experimental site.
**Fig. S2** Climate in Caxiuanã, Brazil.
**Fig. S3** Graphical representation and observed the stem diameter increment.
**Fig. S4** Size structure of the tree community.
**Fig. S5** Relationship between the initial diameter at the breast height.
**Fig. S6** Relationship between small tree stem increment and small tree basal area.
**Table S1** Number of individuals sampled for functional trait measurements.
**Table S2** Pearson correlation coefficients between the 16 functional traits and three PCA axes.
**Table S3** Effects of functional trait variation axes on small tree growth rates.
**Table S4** Effects of tree density on small tree stem increment at the subplot scale.
**Table S5** Pearson correlation (r) between small tree (DBH 1–10 cm) vs large tree (DBH > 10 cm).
Please note: Wiley is not responsible for the content or functionality of any Supporting Information supplied by the authors. Any queries (other than missing material) should be directed to the *New Phytologist* Central Office.

## Data Availability

The data and code required to reproduce the analyses are available at doi: 10.5281/zenodo.17879009.
