# Supplementary material for: Small understory trees increase growth following sustained drought in the Amazon
Source: New Phytol. 2026 Jan 2;249(6):2787–99. doi: 10.1111/nph.70873 (PMC12917452; doi:10.1111/nph.70873)
Supplement: Supplementary file 1 — Fig. S1 Illustration of the experimental site. Fig. S2 Climate in Caxiuanã, Brazil. Fig. S3 Graphical representation and observed the stem diameter increment. Fig. S4 Size structure of the tree community. Fig. S5 Relationship between the initial diameter at the breast height. Fig. S6 Relationship between small tree stem increment and small tree basal area. Table S1 Number of individuals sampled for functional trait measurements. Table S2 Pearson correlation coefficients between the 16 functional traits and three PCA axes. Table S3 Effects of functional trait variation axes on small tree growth rates. Table S4 Effects of tree density on small tree stem increment at the subplot scale. Table S5 Pearson correlation (r) between small tree (DBH 1–10 cm) vs large tree (DBH > 10 cm). Please note: Wiley is not responsible for the content or functionality of any Supporting Information supplied by the authors. Any queries (other than missing material) should be directed to the New Phytologist Central Office. [file NPH-249-2787-s001.pdf]

New Phytologist Supporting Information

Article title: Small understory trees increase growth following sustained drought in the Amazon

Authors: Mateus C. Silva, David C. Bartholomew, André L. Giles, Paulo R. L. Bittencourt, Pablo Sanchez-Martinez, Lion R. Martius, Vanessa N. Rodrigues, Rachel Selman, João P. Reis, Grazielle S. Teodoro, Rafael S. Oliveira, Oliver Binks, Maurizio Mencuccini, João A. Silva Junior, Antonio C. L. da Costa, Patrick Meir, Lucy Rowland

Article acceptance date: 11 December 2025

Table S1. Number of individuals sampled for functional trait measurements in the Control and Throughfall Exclusion (TFE) plots.

**Genus**	**Species**	**Control**	**TFE**
Duguetia	Duguetia cadaverica Huber	2	1
Eschweilera	Eschweilera coriacea (DC.) S.A.Mori	3	3
Inga	Inga heterophylla Willd.	4	3
Iryanthera	Iryanthera laevis Markgr.	1	2
Licania	Licania canescens Benoist	2	3
Licania	Licania egleri Prance	1	0
Licania	Licania octandra (Hoffmanns. ex Roem. & Schult.) Kuntze	1	1
Manilkara	Manilkara elata (Allemão ex Miq.) Monach.	1	2
Minquartia	Minquartia guianensis Aubl.	5	1
Mouriri	Mouriri brachyanthera Ducke	3	3
Ocotea	Ocotea caudata (Nees) Mez	3	0
Protium	Protium altissimum (Aubl.) Marchand	4	0
Protium	Protium apiculatum Swart	1	0
Protium	Protium krukovii Swart	2	0
Protium	Protium stevensonii (Standl.) Daly	1	3
Protium	Protium trifoliolatum Engl.	3	3
Swartzia	Swartzia racemosa Benth.	3	2
Vouacapoua	Vouacapoua americana Aubl.	3	3
Ocotea	Ocotea canaliculata (Rich.) Mez	0	3

Table S2. Pearson correlation coefficients between the 16 functional traits and three principal component (PC) axes. V_{cmax} corresponds to maximum rate of Rubisco carboxylation; J_{max}, maximum electron transport rate; R_{dark}, leaf dark respiration rate; g_{min}, leaf minimum conductance; N_{mass}, leaf nitrogen content; P_{mass}, leaf phosphorus content; LMA, leaf mass per area; L_{th}, leaf thickness; ρ, wood density; Ψ_{pd}, predawn leaf water potential; Ψ_{md}, midday leaf water potential; P50, water potential at which 50% of conductivity loss; P88, water potential at which 88% of conductivity loss; K_{smax}, maximum xylem-specific hydraulic conductivity; PLC, percentage loss of xylem conductivity; and A_l:A_s, leaf-to-sapwood area ratio. Bold indicates statistically significant predictors ($P < 0.05$). Significance levels: ns or non-significant $P > 0.05$, * $P \leq 0.05$, ** $P \leq 0.01$, *** $P \leq 0.001$.

Trait	Photosynthetic potential (PC1)	Nutrient content (PC2)	Embolism resistance (PC3)
V_{cmax}	**0.79*****	0.06^{ns}	-0.17^{ns}
J_{max}	**0.76*****	0.08^{ns}	0.01^{ns}
R_{dark}	**0.47***	-0.12^{ns}	0.10^{ns}
g_{min}	0.20^{ns}	0.28^{ns}	**0.51****
N_{mass}	0.07^{ns}	**0.80*****	-0.05^{ns}
P_{mass}	-0.14^{ns}	**0.83*****	0.21^{ns}
LMA	**0.61*****	**-0.55****	-0.27^{ns}
L_{th}	**0.64*****	-0.14^{ns}	-0.09^{ns}
ρ	-0.30^{ns}	-0.11^{ns}	-0.08^{ns}
Ψ_{pd}	**-0.60*****	0.05^{ns}	0.18^{ns}
Ψ_{md}	**-0.69*****	-0.16^{ns}	0.04^{ns}
P50	-0.35^{ns}	0.01^{ns}	**-0.85*****
P88	-0.27^{ns}	0.12^{ns}	**-0.84*****
K_{smax}	0.30^{ns}	**0.47****	-0.33^{ns}
PLC	0.20^{ns}	0.09^{ns}	0.03^{ns}
A_l:A_s	0.26^{ns}	**0.54****	-0.30^{ns}

Table S3. Effects of functional trait variation axes on small tree growth rates. Response variables are the species-averaged stem increment rate per plot (Throughfall Exclusion, TFE, *vs*. Control) and the difference in increment rates between shared species across plots (Δstem increment, TFE − Control). Predictors of stem increment were three principal components (PCs) summarising trait covariation across 16 functional traits. For the Δ stem increment, predictors were the differences in species-averaged PC scores between TFE and Control plots. Final models represent the linear models with the lowest Akaike Information Criterion (AIC) from all possible combinations of the three PCs or ΔPCs. PC2 was interpreted as a "Nutrient content" axis and PC3 as a "Embolism resistance" axis. Bold indicates statistically significant predictors ($P < 0.05$). A colon ":" denotes an interaction term.

**Response**	**Predictor**	**Estimate**	**t-value**	***P***
Stem increment	**(Intercept)**	7.77×10^{-2}	5.00	**<0.001**
	Nutrient content	-2.92×10^{-3}	-0.24	0.80
	Embolism resistance	-1.42×10^{-2}	-1.68	0.10
	**$Plot_{TFE}$**	6.99×10^{-2}	2.90	**0.007**
	**Nutrient content:$Plot_{TFE}$**	3.68×10^{-2}	2.28	**0.03**
ΔStem increment	(Intercept)	2.32×10^{-2}	0.92	0.37
	**ΔNutrient content**	6.98×10^{-2}	4.48	**0.001**

Table S4. Effects of tree density on small tree stem increment at the subplot scale. Each model corresponds to a subplot-averaged stem increment. The first two "single size" models show stem increment as a function of plot interacting with either small tree density (DBH 1–10 cm) or large tree density (DBH > 10 cm) expressed in individuals per subplot. The third model is a full model including both small and large tree density under the formula: stem increment ~ (small tree density + large tree density) * plot. Bold indicates statistically significant predictors (P < 0.05). A colon ":" denotes an interaction term. "TFE" stands for Throughfall Exclusion.

**Model**	**Predictor**	**Estimate**	***t*-value**	***P***
Single size (small)	**(Intercept)**	1.01×10^{-1}	2.71	**0.01**
	Small tree density	-6.82×10^{-4}	-0.52	0.60
	**$\mathbf{Plot_{TFE}}$**	1.99×10^{-1}	4.02	**<0.001**
	**Small tree density:$\mathbf{Plot_{TFE}}$**	-6.92×10^{-3}	-2.75	**0.009**
Single size (large)	**(Intercept)**	9.90×10^{-2}	2.43	**0.02**
	Large tree density	-3.54×10^{-3}	-0.46	0.64
	**$\mathbf{Plot_{TFE}}$**	1.50×10^{-1}	3.02	**0.004**
	Large tree density:$Plot_{TFE}$	-6.47×10^{-3}	-0.73	0.46
Full size	**(Intercept)**	1.20×10^{-1}	2.24	**0.03**
	Small tree density	-7.51×10^{-4}	-0.55	0.58
	Large tree density	-3.63×10^{-3}	-0.51	0.60
	**$\mathbf{Plot_{TFE}}$**	1.97×10^{-1}	3.04	**0.004**
	**Small tree density:$\mathbf{Plot_{TFE}}$**	-5.80×10^{-3}	-2.08	**0.04**
	Large tree density:$Plot_{TFE}$	-1.63×10^{-3}	-0.19	0.84

Table S5. Pearson correlation (*r*) between small tree (DBH 1–10 cm) *vs*. large tree (DBH > 10 cm) and density (individuals per subplot) and basal area (m^2 per hectare) calculated for subplots (100 m^2) in the Throughfall Exclusion plot (TFE) and Control plot. *r* corresponds to the Pearson correlation coefficient, and *P* to the *P*-value. "~" denotes the correlation terms.

**Plot**	**Formula**	***r***	***P***
Control	Large tree density ~ small tree density	-0.02	0.91
	Large tree basal area ~ small tree basal area	0.13	0.57
TFE	Large tree density ~ small tree density	0.38	0.11
	Large tree basal area ~ small tree basal area	-0.13	0.59

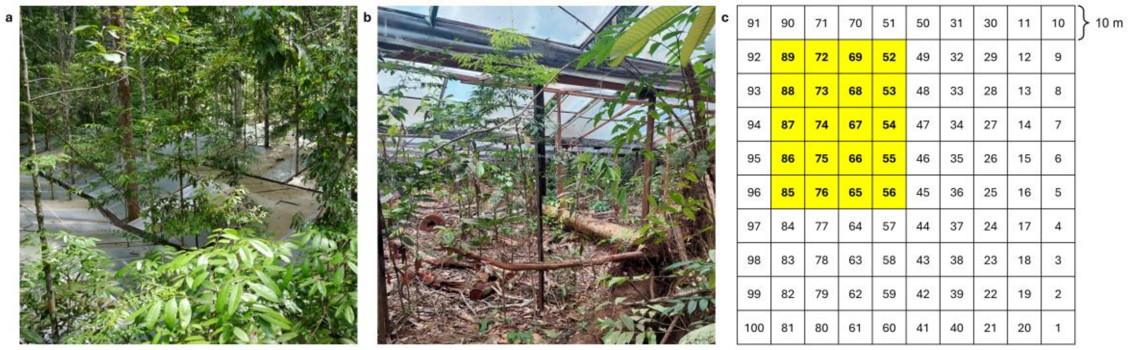

Figure S1. Illustration of the experimental site. View of the Throughfall Exclusion (TFE) plot (**a**) above and (**b**) underneath the rainout panels. (**c**) Graphical representation of the experiment setup. The squares represent the 100 10 × 10 m subplots found in both TFE and Control plots (1 ha each). The sapling (DBH 1–10 cm) community has been monitored in 20 yellow subplots, located at the top-left corner, but avoiding the 1 ha plot margin. The subplot numbering is identical in the TFE and Control plots.

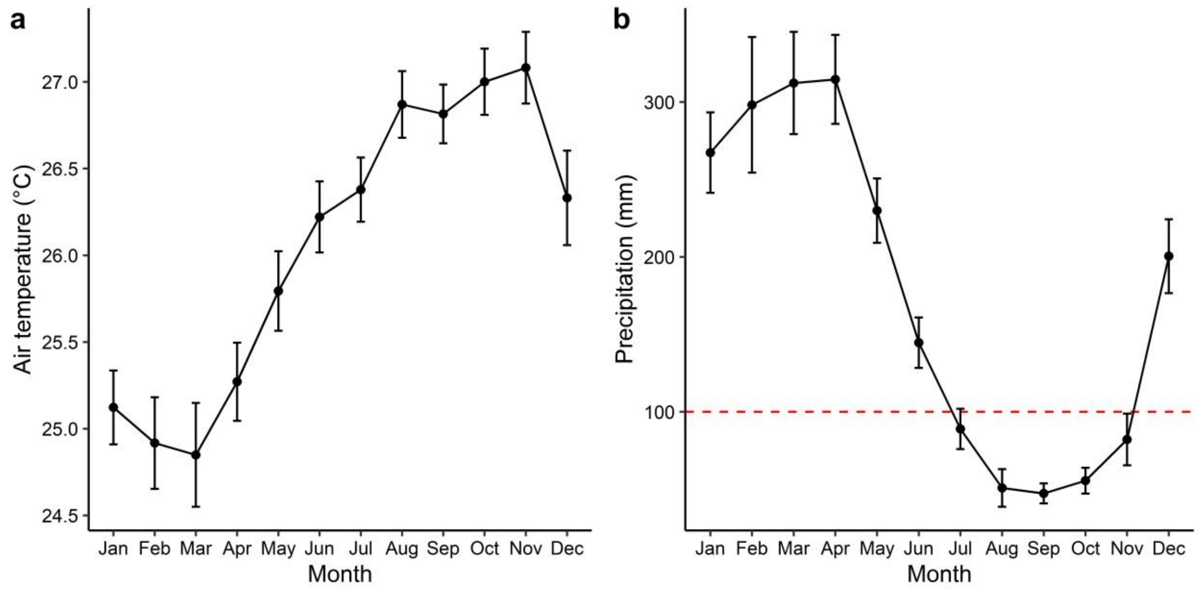

Figure S2. Climate in Caxiuanã, Brazil. (**a**) Mean monthly air temperature and (**b**) cumulative precipitation, averaged from 1996 to 2023, based on data from a meteorological station located atop a 42 m tall tower in the centre of the Control plot. Points represent monthly means, and vertical bars indicate standard errors. In panel **b**, the dashed red line marks the 100 mm precipitation threshold used to define the dry season.

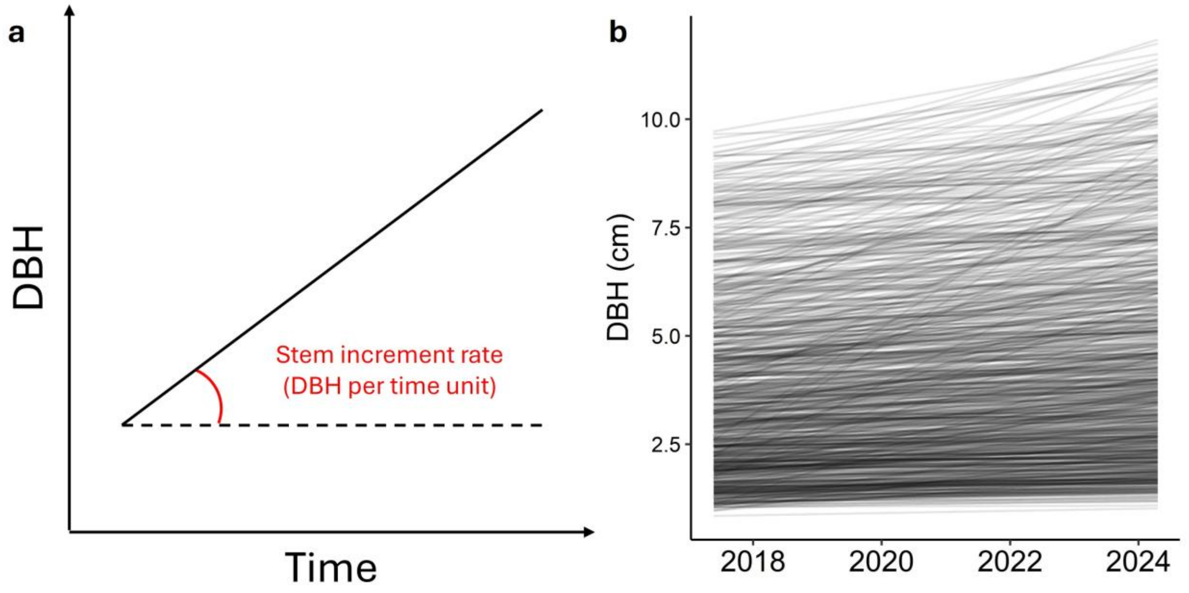

Figure S3. (**a**) Graphical representation and (**b**) observed the stem diameter increment. Note that the increment rate in panel **a** refers to the change in the diameter at the breast height (DBH, 1.3 m) over time (i.e., repeated censuses). Each line in panel **b** corresponds to the fitted regression line of a single individual.

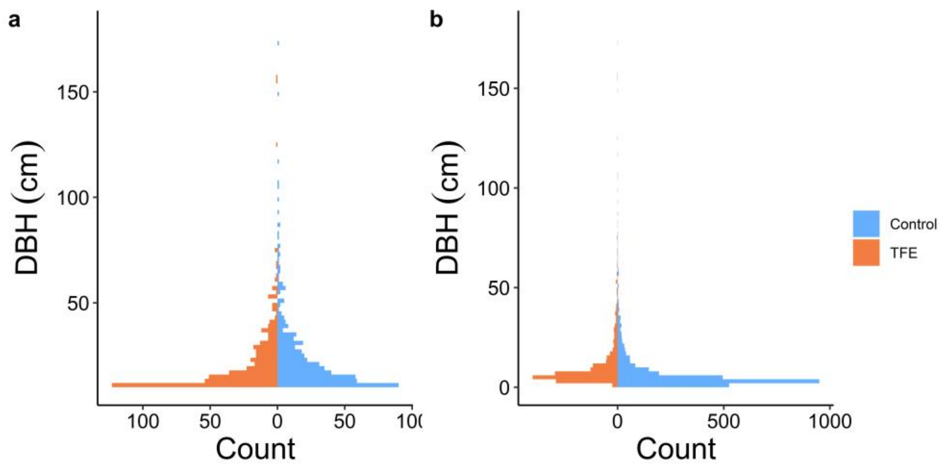

Figure S4. Size structure of the tree community. Mirrored histograms show the number of individuals per size class in the Throughfall Exclusion (TFE) plot (left) and the Control plot (right), considering (**a**) only large trees (DBH > 10 cm) and (**b**) both large and small trees (DBH > 1 cm), with small tree counts scaled to 1 ha.

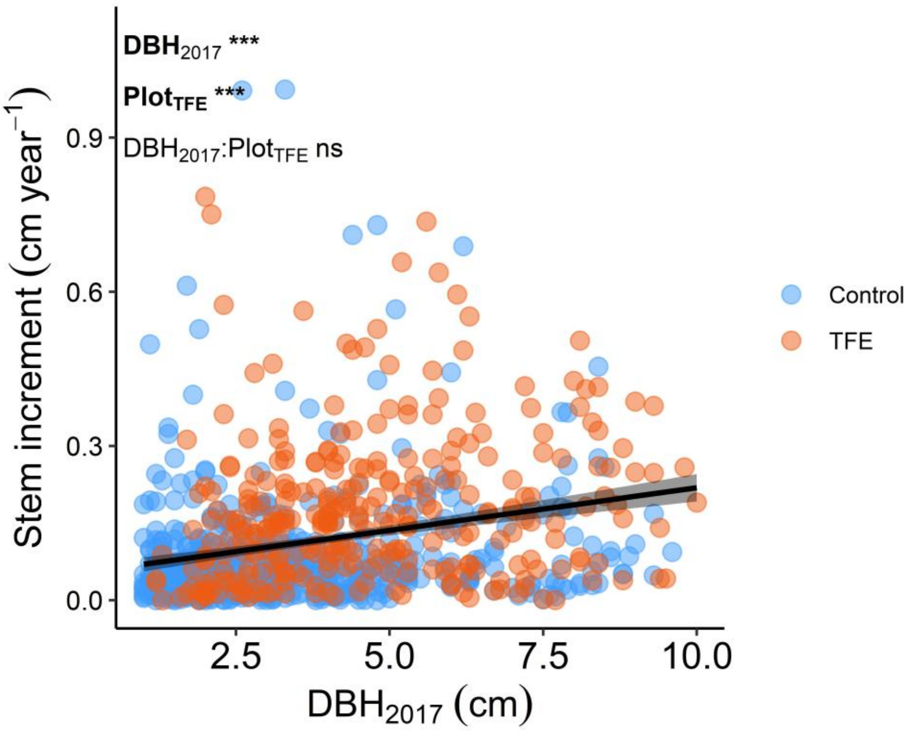

Figure S5. Relationship between the initial diameter at the breast height (DBH_{2017}) measured in the first census (2017) and the stem increment calculated between 2017 and 2024. The significance level in the top-left corner corresponds to linear models fitted to stem increment as a function of DBH_{2017} interacting with plot. "TFE" stands for Throughfall Exclusion and "DBH" to diameter at the breast height. $P < 0.05$ is in bold. ns or non-significant $P > 0.05$, *** $P \leq 0.001$.

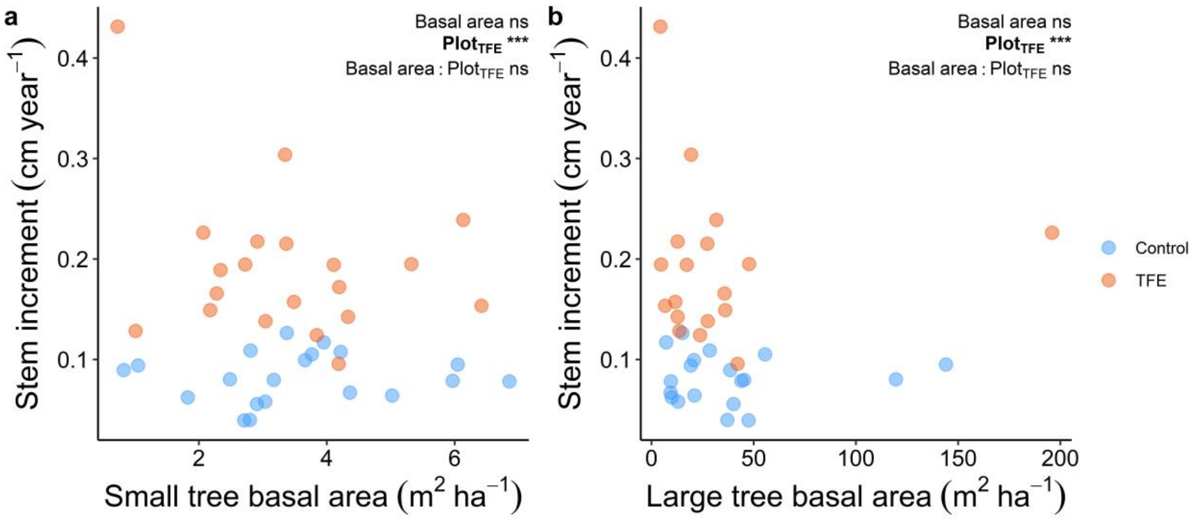

Figure S6. (**a**) Relationship between small tree stem increment and small tree basal area (DBH 1-10 cm) and (**b**) large tree basal area (DBH > 10 cm). Each observation corresponds to a subplot (100 m^2) within the Throughfall Exclusion plot (TFE) and Control plot. Stem increment was averaged per subplot. *P*-values at the top-left corner correspond to the linear models fitted to stem increment as a function of the basal area, plot (TFE), and their interaction. $P < 0.05$ is in bold. Only statistically significant fitted lines are shown. The dashed line marks a stem increment of zero. Significance levels: ns or non-significant $P > 0.05$, *** $P \leq 0.001$.
